# An improved spreadsheet for calculating limb length discrepancy and epiphysiodesis timing using the multiplier method

**DOI:** 10.1007/s11832-016-0754-4

**Published:** 2016-06-29

**Authors:** Gavin Mills, Scott Nelson

**Affiliations:** School of Medicine, Loma Linda University, 11175 Campus St, Loma Linda, CA 92350 USA; Department of Orthopedics, Loma Linda University Medical Center, 11234 Anderson St, Loma Linda, CA 92354 USA

**Keywords:** Spreadsheet, Multiplier method, Limb length discrepancy, Epiphysiodesis

## Abstract

**Purpose:**

The multiplier method is a technique to predict limb length discrepancy (LLD) at maturity in pediatric patients. Various tools have been developed for performing the multiplier calculations to predict LLD and timing of epiphysiodesis. These include multiplier/growth applications (apps) and a spreadsheet which have helped to facilitate LLC calculations in an efficient and easy manner. We have updated the spreadsheet to improve features for making LLD calculations and facilitate pasting data into electronic medical records (EMRs).

**Methods:**

Tools currently in use were critically examined for features that limited their function, created possible sources of error or could be more user-friendly. These features were modified and recreated in an improved Excel spreadsheet that uses patient age, sex, limb lengths, and previous lengthening surgeries as inputs to predict LLD at maturity and offer options for timing of epiphysiodesis for both congenital and developmental LLD. Our multiplier spreadsheet function was then compared to manual calculations and other multiplier tools for accuracy and ease of use.

**Results:**

Our spreadsheet accurately calculates LLD at maturity and timing of epiphysiodesis when compared to other methods. It contains a function to calculate predicted leg lengths after previous lengthenings, and concise single-page worksheets for developmental LLD, congenital LLD, and height prediction.

**Conclusions:**

This spreadsheet was developed to provide a more efficient and user-friendly method of calculating LLD at maturity and timing of epiphysiodesis. It can easily be pasted into the EMR for ease of documentation. We recommend this method for both clinical practice and educational use.

**Electronic supplementary material:**

The online version of this article (doi:10.1007/s11832-016-0754-4) contains supplementary material, which is available to authorized users.

## Background

The multiplier method was first developed by Paley et al. in 2000 to predict limb length discrepancy (LLD) at skeletal maturity and the timing of epiphysiodesis [1]. They looked at a number of different populations to confirm that for a given chronological age and sex, the ratio of a patient’s bone length at maturity to current bone length remains the same across different races, anthropologic eras, and height percentiles. The ratios were made into a table of multipliers for a given sex and age (Table 1). Validating studies have further supported the use of the multiplier method and favorably compared it to other methods as an accurate and reliable tool for clinical application [2–6]. The practicality of this method was greatly enhanced by the creation of the Multiplier application (app) and the Paley Growth (PG) app. They both use the multiplier method in an iOS and Android interface to facilitate the calculations for clinical use. They contain a number of features used in different clinical scenarios to include calculations for upper and lower extremity LLD, timing of epiphysiodesis, height and growth charts and information regarding other growth disorders. The formulae within the apps are derived from those of the original formulae from Paley et al. Additionally, Sanders et al. published a Microsoft Excel spreadsheet that, apart from the obvious differences in interface, functions similarly to the Multiplier app and the PG app [7]. It calculates predicted lower extremity LLD and timing of epiphysiodesis using the multiplier method formulae and tables for both congenital and developmental LLD. Our goal was to improve upon this spreadsheet by making a more user-friendly interface that could be cleanly pasted into electronic medical record (EMR) progress notes and to add several useful features.

**Table 1:** Age- and gender-specific multipliers from the Multiplier app [17]

Age	Boys	Girls	Age	Boys	Girls	Age	Boys	Girls	Age	Boys	Girls
0.00	5.08	4.63	4.25	1.96	1.79	8.50	1.43	1.30	12.83	1.14	1.04
0.08	4.93	4.49	4.33	1.94	1.77	8.58	1.42	1.29	12.92	1.13	1.03
0.17	4.77	4.35	4.42	1.93	1.76	8.67	1.41	1.28	13.00	1.13	1.03
0.25	4.62	4.22	4.50	1.91	1.75	8.75	1.40	1.28	13.08	1.13	1.03
0.33	4.47	4.08	4.58	1.90	1.73	8.83	1.40	1.27	13.17	1.12	1.03
0.42	4.31	3.94	4.67	1.88	1.72	8.92	1.39	1.27	13.25	1.12	1.02
0.50	4.16	3.80	4.75	1.87	1.70	9.00	1.38	1.26	13.33	1.11	1.02
0.58	4.01	3.66	4.83	1.85	1.69	9.08	1.37	1.25	13.42	1.11	1.02
0.67	3.85	3.52	4.92	1.84	1.67	9.17	1.37	1.25	13.50	1.11	1.02
0.75	3.70	3.39	5.00	1.82	1.66	9.25	1.36	1.24	13.58	1.10	1.01
0.83	3.55	3.25	5.08	1.81	1.65	9.33	1.36	1.24	13.67	1.10	1.01
0.92	3.39	3.11	5.17	1.80	1.64	9.42	1.35	1.23	13.75	1.09	1.01
1.00	3.24	2.97	5.25	1.78	1.62	9.50	1.35	1.23	13.83	1.09	1.01
1.08	3.19	2.92	5.33	1.77	1.61	9.58	1.34	1.22	13.92	1.08	1.00
1.17	3.13	2.87	5.42	1.76	1.60	9.67	1.33	1.21	14.00	1.08	1.00
1.25	3.08	2.83	5.50	1.75	1.59	9.75	1.33	1.21	14.08	1.08	–
1.33	3.02	2.78	5.58	1.73	1.57	9.83	1.32	1.20	14.17	1.07	–
1.42	2.97	2.73	5.67	1.72	1.56	9.92	1.32	1.20	14.25	1.07	–
1.50	2.92	2.68	5.75	1.71	1.55	10.00	1.31	1.19	14.33	1.07	–
1.58	2.86	2.63	5.83	1.70	1.54	10.08	1.30	1.19	14.42	1.06	–
1.67	2.81	2.58	5.92	1.68	1.52	10.17	1.30	1.18	14.50	1.06	–
1.75	2.75	2.54	6.00	1.67	1.51	10.25	1.29	1.18	14.58	1.06	–
1.83	2.70	2.49	6.08	1.66	1.50	10.33	1.29	1.17	14.67	1.05	–
1.92	2.64	2.44	6.17	1.65	1.50	10.42	1.28	1.17	14.75	1.05	–
2.00	2.59	2.39	6.25	1.65	1.49	10.50	1.28	1.16	14.83	1.05	–
2.08	2.56	2.36	6.33	1.64	1.48	10.58	1.27	1.16	14.92	1.04	–
2.17	2.53	2.33	6.42	1.63	1.48	10.67	1.26	1.15	15.00	1.04	–
2.25	2.50	2.31	6.50	1.62	1.47	10.75	1.26	1.15	15.08	1.04	–
2.33	2.47	2.28	6.58	1.61	1.46	10.83	1.25	1.14	15.17	1.04	–
2.42	2.44	2.25	6.67	1.60	1.46	10.92	1.25	1.14	15.25	1.03	–
2.50	2.41	2.22	6.75	1.60	1.45	11.00	1.24	1.13	15.33	1.03	–
2.58	2.38	2.19	6.83	1.59	1.44	11.08	1.24	1.13	15.42	1.03	–
2.67	2.35	2.16	6.92	1.58	1.44	11.17	1.23	1.12	15.50	1.03	–
2.75	2.32	2.14	7.00	1.57	1.43	11.25	1.23	1.12	15.58	1.02	–
2.83	2.29	2.11	7.08	1.56	1.42	11.33	1.22	1.11	15.67	1.02	–
2.92	2.26	2.08	7.17	1.55	1.41	11.42	1.22	1.11	15.75	1.02	–
3.00	2.23	2.05	7.25	1.55	1.41	11.50	1.21	1.10	15.83	1.02	–
3.08	2.21	3.03	7.33	1.54	1.40	11.58	1.21	1.10	15.92	1.01	–
3.17	2.19	2.01	7.42	1.53	1.39	11.67	1.20	1.09	16.00	1.01	–
3.25	2.17	2.00	7.50	1.52	1.38	11.75	1.20	1.09	16.08	1.01	–
3.33	2.15	1.98	7.58	1.51	1.37	11.83	1.19	1.08	16.17	1.01	–
3.42	2.13	1.96	7.67	1.50	1.36	11.92	1.19	1.08	16.25	1.01	–
3.50	2.12	1.94	7.75	1.50	1.36	12.00	1.18	1.07	16.33	1.01	–
3.58	2.10	1.92	7.83	1.49	1.35	12.08	1.18	1.07	16.42	1.01	–
3.67	2.08	1.90	7.92	1.48	1.34	12.17	1.17	1.06	16.50	1.01	–
3.75	2.06	1.89	8.00	1.47	1.33	12.25	1.17	1.06	16.58	1.00	–
3.83	2.04	1.87	8.08	1.46	1.32	12.33	1.16	1.06	16.67	1.00	–
3.92	2.02	1.85	8.17	1.46	1.32	12.42	1.16	1.05	16.75	1.00	–
4.00	2.00	1.83	8.25	1.45	1.31	12.50	1.16	1.05	16.83	1.00	–
4.08	1.99	1.82	8.33	1.44	1.31	12.58	1.15	1.05	16.92	1.00	–
4.17	1.97	1.80	8.42	1.43	1.30	12.67	1.15	1.04	17.00	1.00	–

## Discussion

**Fig. 1:**
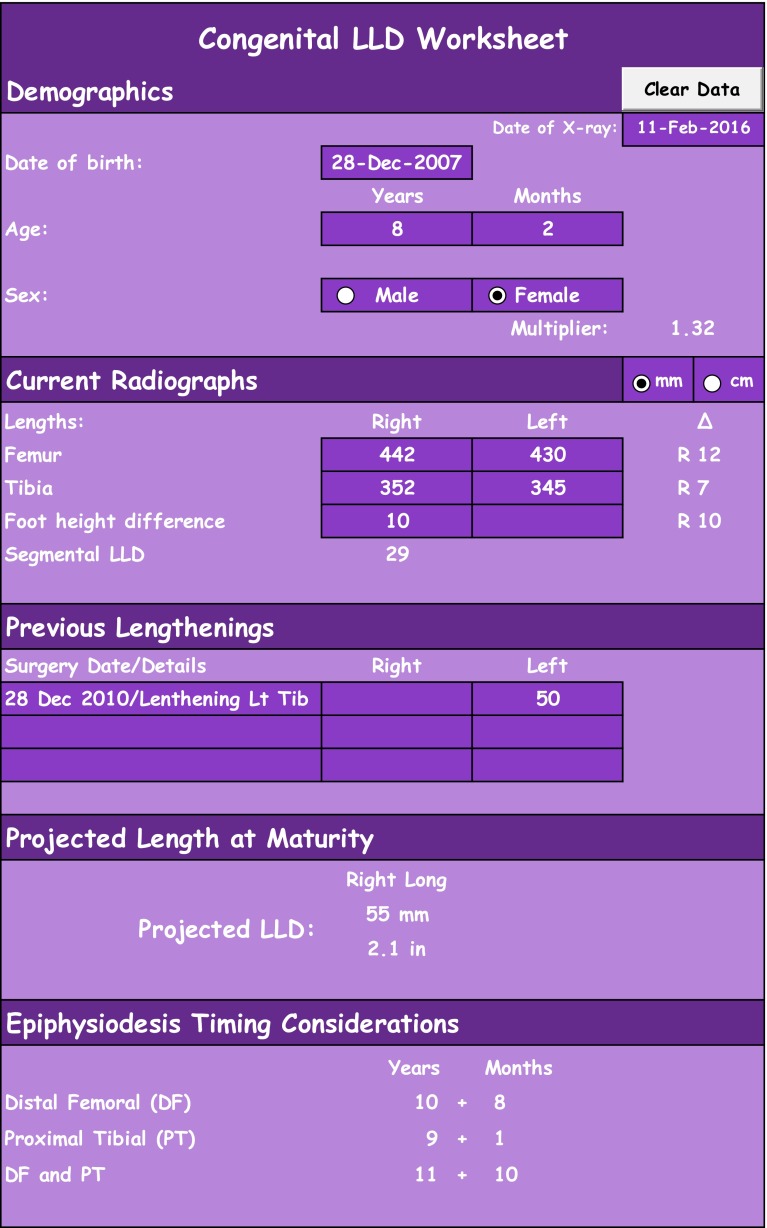
Congenital LLD worksheet

**Fig. 2:**
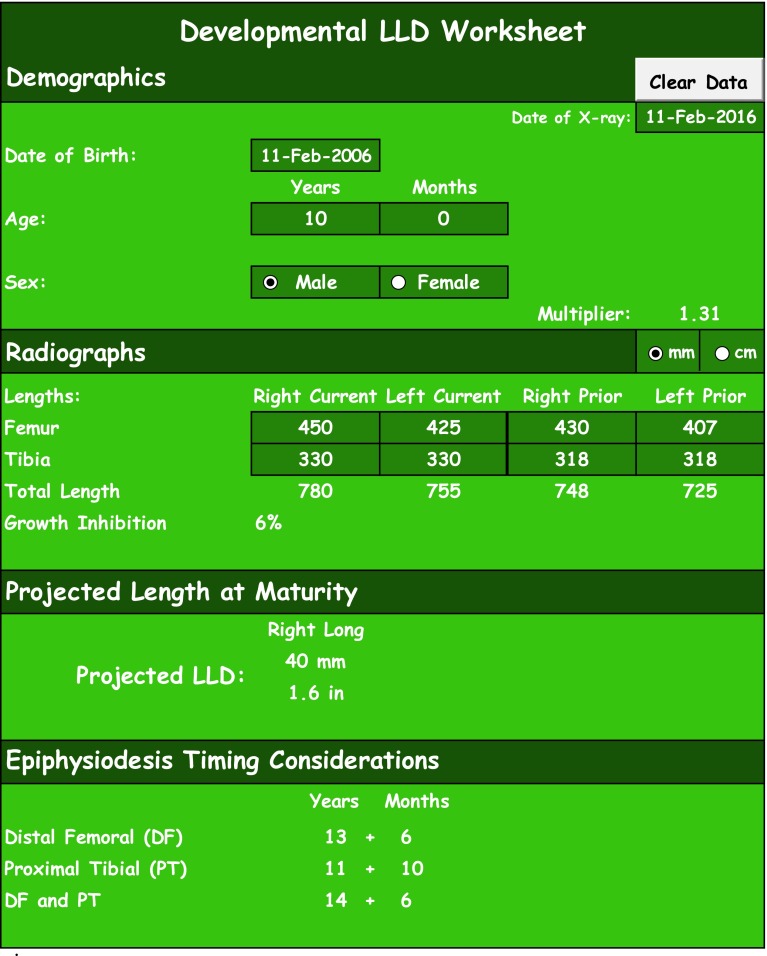
Developmental LLD worksheet

This update to the spreadsheet by Sanders et al. enables the user to enter previous lengthening surgeries when calculating LLD at skeletal maturity and timing of epiphysiodesis (Figs. 1, 2) [7]. It also provides the option to enter foot height, which can be a significant contributor to congenital causes of LLD. The calculations are simplified into separate worksheets for congenital and developmental LLD which helps to differentiate these clinical scenarios and provides a clean-looking datasheet for conveniently pasting into an EMR. The date of the calculations is clearly placed at the top of the datasheet to avoid confusion when copying and updating notes in the EMR. This also allows for multiple worksheets from different dates to be copied into a single progress note so the clinician can see trends, know lengthening history, and better predict LLD. Like the first edition of this worksheet, the process of predicting LLD and appropriate timing of epiphysiodesis is simplified into a single step from the two-step process required by both of the apps. Additionally, a separate tab is included to predict adult height at skeletal maturity to assist clinicians when parents ask about their child’s growth potential. This table is similar to the adult height calculators found in both of the apps using the multiplier method for predicting adult height [8].

The formulae used in our spreadsheet were derived from the multiplier method developed by Paley et al. [1, 9]. The use of the multiplier method for congenital discrepancy assumes a Shapiro type 1 growth pattern where the ratio of growth in the short limb to that of the long limb does not change over time [10]. The congenital LLD table is used for congenital short femur, fibular hemimelia, hemihypertrophy, hemiatrophy, and posteriomedial bowing of the tibia [1]. The developmental LLD table is used for Ollier disease, poliomyelitis, growth arrest, or for post-traumatic discrepancies [1]. These formulae contained minor differences when compared to the Sanders spreadsheet. The growth constants for each physis in the Multiplier app and PG app are 0.71 for the distal femoral physis, 0.57 for the proximal tibial physis, and 0.67 for both the proximal tibial and distal femoral physis together. The Sanders worksheet uses 0.71 for the distal femoral physis, 0.54 for the proximal tibial physis, and uses both the individual distal femoral and proximal tibial constants when calculating timing of epiphysiodesis at both physes. We used the same growth constants as the Multiplier and PG app as published in ‘Principles of Deformity Correction’ in reference to Anderson et al. [9, 11].

Additionally, the user should recognize that the application of our worksheets must be in the appropriate clinical context similar to the multiplier method. A number of studies suggest that the multiplier method, which is based on the patient’s chronological age, has a limited scope and should be applied judiciously when compared to using the radiographic skeletal age. For example, Sanders et al. demonstrated that using the multiplier method with chronologic age is superior to using it with skeletal age for children who have not yet reached their adolescent growth spurt, but that the reverse is true once the child reaches their adolescent growth spurt [6]. For a given population, the chronological age is equal to skeletal age, thus the use of the chronological age is most accurate for children who reach the adolescent growth spurt closest to the average age of onset. The normal pubertal growth spurt lasts for 4 years, has a midpoint of age 12 for girls and age 14 for boys, but can have a normal variation of 4 years [12]. In the context of a large discrepancy between chronologic and skeletal age, Paley et al. have shown that accurate predictions can be still obtained when skeletal age is used in the multiplier method calculations [2]. Given that several studies demonstrate a widening discrepancy between chronological and skeletal age at the onset of the adolescent growth spurt, we suggest using skeletal age in our spreadsheets after 10 years of age in accordance with the accepted standard [2, 9, 13]. Several validated methods of calculating skeletal age that are currently in use include the Greulich and Pyle atlas, Tanner–Whitehouse method, Dimeglio’s method, and the shorthand bone age assessment by Heyworth et al. [5, 14–16].

### Instructions for use

The user only enters data into the dark purple, dark green, or dark blue cells on the worksheets. Any unit of length can be used in the LLD tables, but for accurate cm-inch or mm-inch conversion automatically displayed in the table, the entered data must be in either cm or mm. The ‘Clear Data’ button will clear all the entered data in the table and reset the date. The user cannot undo this function with Excel ‘Undo’ button or Ctrl + Z. Additionally, the user should note that ‘Epiphysiodesis Timing Considerations’ are simply calculating the age that an epiphysiodesis could be performed to correct for the projected LLD given the entered data. Clinical judgment must still be used to determine which epiphysiodesis would be most appropriate given the clinical scenario.

#### Instructions for LLD tables

- Begin by entering the date on which the X-ray was taken. The spreadsheet displays today’s date by default.
- Enter the patient’s date of birth. The spreadsheet calculates the patient’s age with the difference between the date of X-ray and the patient’s date of birth. If no date of birth is entered, the user may enter the patient’s age and the calculations thereafter will be based on the entered patient age.
- Select the patient’s sex.
- Select the unit of measurement, cm or mm (only changes the conversion to inches).
- Enter the lengths of bilateral femurs or bilateral tibias or foot heights^1^ or all three.
  There is no known foot height multiplier (there is a known foot length multiplier) but we know that foot height difference increases with growth and have thus applied the lower extremity multiplier to this parameter. Due to foot height differences that are relatively small, a slightly discrepant foot height multiplier is unlikely to significantly change predicted LLD values.
- In the Congenital LLD Worksheet only:
  - ∆*R*/*L* shows the current bone length differences and *R* or *L* depending on which side is longer.
  - Segmental LLD^2^ shows the value of the current leg length discrepancy on whichever side is longer.
  - Previous lengthening provides cells to enter the amount of lengthening on either of the lower extremities and a column to note the details of the surgery.

For the sake of simplicity, pelvic height differences are not programmable in this worksheet, but if present, should be taken into account when determining the goals of leg length equalization.

## Examples

A female aged 8 years and 2 months with a congenital LLD and a date of birth of 28 December 2007 receives radiographs of bilateral lower extremities on 11 Feb 2016 that reveal the following measurements: right femur 442 mm; left femur 430 mm; right tibia 352 mm; left tibia 345 mm; relative foot height difference 10 mm right side tall. Additionally she received a 50 mm lengthening of the left tibia at 3 years of age (Fig. 1).

A male aged 10 years with a developmental LLD and a date of birth of 11 February 2006 receives radiographs of bilateral lower extremities on 11 February 2016 that reveal the following measurements: right current femur 450 mm; left current femur 425 mm; right prior femur 430 mm; left prior femur 407 mm; right current tibia 330 mm; left current tibia 330 mm; right prior tibia 318 mm; left prior tibia 318 mm (Fig. 2).

## Summary

The function of our spreadsheet was compared to manual calculations using the multiplier method, the Multiplier app, the PG app and the Sanders spreadsheet, and appeared to be comparably accurate. This tool provides a concise datasheet that can be placed on the desktop of clinic workstations and allows the multiplier calculations to be easily copied into the medical record. This method is useful for both clinical practice and educational applications.

### Electronic supplementary material

Below is the link to the electronic supplementary material.

Supplementary material 1 (XLS 2559 kb)

## Appendix

*M*_ε_ = multiplier at age of epiphysiodesis

*κ*_F_ = 0.71 for the distal femur

*κ*_T_ = 0.57 for the proximal tibia

*κ*_T + F_ = 0.67 for the femur and tibia together

*B*_L_ = length of long bone or limb

*B*_S_ = length of short bone or limb

*P* = prior lengthening

∆ = age-specific length discrepancy

*L* = age-specific length

*M* = age- and gender-specific multiplier (obtained from multiplier table, Table 1)

Δ_*m*_ = length discrepancy at maturity

*H* = current height

*H*_m_ = height at maturity

Congenital LLD at skeletal maturity:

$$\Delta_{\text{m}} = \Delta \times M$$

or

$$\Delta_{\text{m}} = M \times \left( {\Delta + P} \right) - P$$

$$\Delta = \left( {\frac{{\Delta_{\text{m}} + P}}{M}} \right) - P.$$

Developmental LLD at skeletal maturity:

$$\Delta_{\text{m}} = \Delta + i \times G$$

$${\text{Growth inhibition }} = i = \frac{{1 - (B_{\text{S}} - B^{\prime}_{\text{S}} )}}{{B_{\text{L}} - B^{\prime}_{\text{L}} }}$$

$${\text{Growth remaining}} = G = L(M - 1).$$

Leg length at skeletal maturity:

$$L_{\text{m}} = L \times M.$$

Timing of epiphysiodesis of the proximal tibial physis:

use current *L* and *M*

$$M_{\varepsilon } = \frac{{L_{\text{T}} \times M}}{{L_{\text{T}} \times M - \frac{{\Delta_{\text{mT}} + \Delta_{\text{mF}} }}{{\kappa_{\text{T}} }}}}.$$

Timing of epiphysiodesis of the distal femoral physis:

use current *L* and *M*

$$M_{\varepsilon } = \frac{{L_{\text{F}} \times M}}{{L_{\text{F}} \times M - \frac{{\Delta_{\text{mT}} + \Delta_{\text{mF}} }}{{\kappa_{\text{F}} }}}}.$$

Timing of epiphysiodesis of both the proximal tibial and distal femoral physis:

use current *L* and *M*

$$M_{\varepsilon } = \frac{{(L_{\text{T}} + L_{\text{F}} ) \times M}}{{(L_{\text{T}} + L_{\text{F}} ) \times M - \frac{{\Delta_{\text{mT}} + \Delta_{\text{mF}} }}{{\kappa_{{{\text{T}} + {\text{F}}}} }}}}.$$

Adult height prediction:

$$H_{\text{m}} = H \times M.$$

## Footnotes

1 Depending on the X-ray technique, it is often impossible to measure actual foot height in which case we just measure the difference between the right and left tibial plafond height and enter this number on the side of the taller foot. It is also important when determining foot height that any lifts are accurately documented and subtracted out, that the patient is weight-bearing equally on both lower extremities, and that there are no unrecognized equinus contractures. There is no known foot height multiplier (there is a known foot length multiplier) but we know that foot height difference increases with growth and have thus applied the lower extremity multiplier to this parameter. Due to foot height differences that are relatively small, a slightly discrepant foot height multiplier is unlikely to significantly change predicted LLD values.

2 We introduce the term ‘Segmental LLD’ to refer to the LLD that is measured between the long bone and foot segments of the lower extremities. This is sometimes different than overall femoral head height difference (sometimes called global difference) which can be affected by coronal plane deformities (genu varum/valgum). A knee flexion contracture would have an equal effect on segmental bone lengths as well as femoral head height (global) difference on measurements taken from the anteroposterior radiograph. We always examine lateral views on our EOS images to detect this potential source of error.
